# Draft Genome Sequence of a Sequence Type 398 Methicillin-Resistant *Staphylococcus aureus* Isolate from a Danish Dairy Cow with Mastitis

**DOI:** 10.1128/genomeA.00492-17

**Published:** 2017-06-08

**Authors:** Troels Ronco, Marc Stegger, Karl Pedersen

**Affiliations:** a National Veterinary Institute, Technical University of Denmark, Frederiksberg, Denmark; b Department of Bacteria, Parasites and Fungi, Statens Serum Institut, Copenhagen, Denmark

## Abstract

Livestock-associated (LA) methicillin-resistant *Staphylococcus aureus* (MRSA) strains of sequence type 398 (ST398) colonize both humans and various livestock species. In 2016, an ST398 LA-MRSA isolate (Sa52) was collected from a Danish dairy cow with mastitis, and here, we report the draft genome sequence of strain Sa52.

## GENOME ANNOUNCEMENT

*Staphylococcus aureus* is a notorious opportunistic pathogen that infects both humans and animals and constitutes a major cause of bovine mastitis (1). In Denmark, livestock-associated (LA) methicillin-resistant *S. aureus* (MRSA) strains of sequence type 398 (ST398) primarily colonize pigs but are now also increasingly causing human infections (2, 3). The ST398 lineage has spread to various livestock species and has been detected in European dairy cattle with mastitis and in bulk-tank milk (1). However, there are hitherto no reports on clinical ST398 MRSA isolates from Danish dairy herds or Danish cattle in general. Here, we present a draft genome sequence of the first reported LA-MRSA isolate (Sa52) of ST398 collected in 2016 from a Danish dairy cow with mastitis.

The Nextera XT DNA kit (Illumina) was used to prepare libraries of fragmented DNA for paired-end sequencing, with an average read length of 2 × 150 bp, applying Illumina’s NextSeq platform. The quality of raw reads was analyzed in FastQC 0.11.5 and *de novo* assembly performed in CLC bio’s Genomics Workbench 10.0. The total assembly and *N*_50_ size were determined to be 2.8 Mb and 34.4 kb, respectively. An average coverage of 103×, 145 contigs, and a G+C content of 32.8% were obtained. Gene annotation was performed using NCBI’s Prokaryotic Genome Automatic Annotation Pipeline (PGAAP) (4) and resulted in a total of 2,878 genes, of which 2,735 were protein-coding sequences (CDSs).

Multilocus sequence typing using PubMLST (5) showed that Sa52 was from ST398. Virulence and resistance genes were identified using ResFinder 2.1 (6) and Virulence Finder 1.5 (7), respectively, whereas the BLASTN 2.2.30+ (8) implementation in CLC bio’s Genomics Workbench was applied for further identification of genomic content. The *mecA* gene and a wide range of other resistance genes (*blaZ*, *ermB, lnuB*, *norA*, *spc*, *tetK*, and* tetM*) were found. The staphylococcal cassette chromosome *mec* element (SCC*mec*) V(5C2&5)c (9) was entirely conserved, and PHASTER (10) identified an intact phage of 45.6 kb, StauST398-2 (GenBank accession no. NC_021323). Virulence genes (*aur*, *cap*, *fib, hla, hlb, hlg, icaD*, and* nuc*) that previously have been found in *S. aureus* strains from cattle with mastitis and in bulk-tank milk were detected (11, 12). Last, a single *hsdM* gene from a type 1 restriction-modification (RM) system was identified via Restriction-Modification Finder 1.1 (http://cge.cbs.dtu.dk). Since no *hsdS* genes were present, it indicated that Sa52 did not carry any functional RM systems, which means that foreign DNA is easily taken up (13).

The ST398 LA-MRSA lineage has disseminated to both humans and various livestock species, and here, we report the first case, to our knowledge, of a clinical isolate of this lineage collected from a Danish dairy cow with mastitis. For this reason, we recommend monitoring of clinical infections in cattle and/or bulk-tank milk samples for MRSA to follow any potential development in the distribution and frequency of LA-MRSA.

### Accession number(s).

This whole-genome shotgun project has been deposited in DDBJ/ENA/GenBank under the accession number NBNF00000000. The version described in this paper is the first version.
